# Supplementary material for: Polyketide synthase gene clusters in the ericoid mycorrhizal fungus Oidiodendron maius: genome-wide prediction, expression profiling, and functional characterization of knockout mutants
Source: Front Plant Sci. 2026 Sep 16;17:1924430. doi: 10.3389/fpls.2026.1924430 (PMC13623903; doi:10.3389/fpls.2026.1924430)
Supplement: Supplementary file 1 [file SupplementaryFile1.zip › Supplementary Materials/Supplementary Folder 1.zip!/Oidma1_AssemblyScaffolds_Repeatmasked/knownclusterblast/cluster52/jgi.p_Oidma1_106943_mibig_hits.html]

| MIBiG Protein | Description | MIBiG Cluster | MiBiG Product | % ID | % Coverage | BLAST Score | E-value |
| --- | --- | --- | --- | --- | --- | --- | --- |
| BAE56588.1 |  | BGC0001123 | Aspirochlorine\_biosynthetic\_gene\_cluster | 59 | 54.1200406918 | 641 | 1.6e-183 |
| EAQ86388.1 | hypothetical\_protein | BGC0001405 | Chaetoviridin\_/\_chaetomugilin\_biosynthetic\_gene\_cluster | 55 | 54.4252288911 | 583 | 4e-166 |
| AGO59046.1 | PtaM | BGC0000121 | Pestheic\_acid\_biosynthetic\_gene\_cluster | 51 | 54.9338758901 | 537 | 2.5e-152 |
| ADI24948.1 | GsfI | BGC0000070 | Griseofulvin\_biosynthetic\_gene\_cluster | 47 | 54.3234994914 | 490 | 4.6e-138 |
| ACD39771.1 | non-heme\_halogenase | BGC0000134 | Radicicol\_biosynthetic\_gene\_cluster | 48 | 54.4252288911 | 490 | 4.6e-138 |
| AEM76785.1 | putative\_halogenase | BGC0001401 | Melleolides\_biosynthetic\_gene\_cluster | 46 | 54.5269582909 | 475 | 1.5e-133 |
| ALT31850.1 | ArmH3 | BGC0001401\_c3 | Melleolides\_biosynthetic\_gene\_cluster | 47 | 50.4577822991 | 456 | 7.3e-128 |
| AEM76786.1 | putative\_halogenase | BGC0001401\_c2 | Melleolides\_biosynthetic\_gene\_cluster | 44 | 55.2390640895 | 441 | 3.2e-123 |
| ALT31851.1 | ArmH4 | BGC0001401\_c4 | Melleolides\_biosynthetic\_gene\_cluster | 45 | 55.0356052899 | 437 | 3.5e-122 |
| ALT31852.1 | ArmH5 | BGC0001401\_c5 | Melleolides\_biosynthetic\_gene\_cluster | 43 | 54.018311292 | 428 | 2.1e-119 |
| ADE22292.1 | putative\_non-heme\_halogenase | BGC0000279 | Xantholipin\_biosynthetic\_gene\_cluster | 37 | 42.522889115 | 264 | 4.1e-70 |
| ABM21576.1 | CrpH | BGC0000975 | Cryptophycin\_biosynthetic\_gene\_cluster | 37 | 39.5727365209 | 264 | 5.3e-70 |
| AMK51270.1 | Hex13 | BGC0001376 | Hexaricin\_biosynthetic\_gene\_cluster | 35 | 44.3540183113 | 248 | 2.3e-65 |
| CAM34371.1 | putative\_non-heme\_halogenase | BGC0000242 | Lysolipin\_biosynthetic\_gene\_cluster | 36 | 42.522889115 | 244 | 4.4e-64 |
| CAJ46693.1 | tryptophan\_halogenase | BGC0000969 | Chondramide\_biosynthetic\_gene\_cluster | 35 | 44.4557477111 | 229 | 1.5e-59 |
| AAS90060.1 | OmtB | BGC0000009 | Aflatoxin\_biosynthetic\_gene\_cluster | 33 | 36.7243133266 | 221 | 4e-57 |
| AEU17894.1 | putative\_halogenase | BGC0001072 | Simocyclinone\_biosynthetic\_gene\_cluster | 32 | 44.2522889115 | 218 | 3.4e-56 |
| WP\_052390547.1 | halogenase | BGC0001308 | Simocyclinone\_D9\_/\_simocyclinone\_D10\_/\_simocyclinone\_D11 | 32 | 44.2522889115 | 217 | 7.5e-56 |
| ACU71639.1 | tryptophan\_halogenase | BGC0001154 | Cacibiocin\_biosynthetic\_gene\_cluster | 35 | 38.0467955239 | 215 | 2.2e-55 |
| ACH72896.1 | AflO | BGC0000011 | Aflatoxin/sterigmatocystin\_biosynthetic\_gene\_cluster | 31 | 37.2329603255 | 208 | 2.7e-53 |
| ADU85999.1 | putative\_tryptophan\_halogenase | BGC0000165 | Tiacumicin\_B\_biosynthetic\_gene\_cluster | 31 | 38.6571719227 | 193 | 1.2e-48 |
| AEI58871.1 | halogenase | BGC0000455 | Vancomycin\_biosynthetic\_gene\_cluster | 33 | 39.3692777213 | 176 | 1.5e-43 |
| ctg1\_orf12 |  | BGC0001366 | BE-24566B\_/\_zunyimycin\_A\_biosynthetic\_gene\_cluster | 33 | 35.4018311292 | 173 | 1.6e-42 |
| AAK81830.1 | non-heme\_halogenase | BGC0000326 | Complestatin\_biosynthetic\_gene\_cluster | 31 | 40.6917599186 | 172 | 2.1e-42 |
| AAN65237.1 | putative\_halogenase | BGC0000832 | Clorobiocin\_biosynthetic\_gene\_cluster | 33 | 35.4018311292 | 172 | 2.1e-42 |
| CAA76550.1 | halogenase | BGC0000311 | Balhimycin\_biosynthetic\_gene\_cluster | 32 | 39.3692777213 | 172 | 2.8e-42 |
| CAA11780.1 | PCZA361.26 | BGC0000322\_c2 | Chloroeremomycin\_biosynthetic\_gene\_cluster | 32 | 39.3692777213 | 172 | 2.8e-42 |
| AFV52210.1 | aryl-ACP\_halogenase | BGC0000081 | Kedarcidin\_biosynthetic\_gene\_cluster | 32 | 40.2848423194 | 171 | 3.6e-42 |
| AAL06656.1 | alkylhalidase | BGC0000965 | C-1027\_biosynthetic\_gene\_cluster | 30 | 39.2675483215 | 165 | 4.4e-40 |
| ALU98528.1 | tryptophan\_halogenase | BGC0001397 | Lidamycin\_biosynthetic\_gene\_cluster | 30 | 38.4537131231 | 164 | 7.5e-40 |
| AGC09515.1 | FAD\_dependent\_halogenase | BGC0001183 | Lobophorin\_biosynthetic\_gene\_cluster | 31 | 38.5554425229 | 163 | 1.7e-39 |
| CAG15020.1 | halogenase | BGC0000441 | Teicoplanin\_biosynthetic\_gene\_cluster | 30 | 39.4710071211 | 161 | 4.9e-39 |
| CAD91205.1 | halogenase | BGC0000289 | A40926\_biosynthetic\_gene\_cluster | 30 | 38.8606307223 | 161 | 4.9e-39 |
| CAE53362.1 | halogenase | BGC0000440 | Teicoplanin\_biosynthetic\_gene\_cluster | 30 | 39.4710071211 | 161 | 4.9e-39 |
| AGS77325.1 | Halogenase | BGC0001178 | UK-68,597\_biosynthetic\_gene\_cluster | 29 | 39.4710071211 | 160 | 1.4e-38 |
| ABD65950.1 | halogenase | BGC0000341 | Enduracidin\_biosynthetic\_gene\_cluster | 29 | 39.1658189217 | 160 | 1.4e-38 |
| AAM80532.1 | StaI | BGC0000290 | A47934\_biosynthetic\_gene\_cluster | 31 | 37.6398779247 | 159 | 1.9e-38 |
| ABS50488.1 | NapH2 | BGC0000652 | Napyradiomycin\_biosynthetic\_gene\_cluster | 30 | 37.6398779247 | 159 | 2.4e-38 |
| ABS50460.1 | NapH2 | BGC0001079 | Napyradiomycin\_biosynthetic\_gene\_cluster | 30 | 37.6398779247 | 159 | 2.4e-38 |
| ALD82533.1 | halogenase | BGC0001212 | Nannocystin\_biosynthetic\_gene\_cluster | 30 | 38.2502543235 | 155 | 2.7e-37 |
| CAQ43074.1 | halogenase | BGC0000970 | Chondrochloren\_biosynthetic\_gene\_cluster | 27 | 38.7589013225 | 147 | 9.5e-35 |
| ABY66006.1 | halogenase | BGC0001008 | Maduropeptin\_biosynthetic\_gene\_cluster | 29 | 38.7589013225 | 146 | 1.2e-34 |
| AAM80530.1 | StaK | BGC0000290 | A47934\_biosynthetic\_gene\_cluster | 29 | 37.1312309257 | 144 | 6.2e-34 |
| ADM46362.1 | halogenase | BGC0000106 | Naphthomycin\_biosynthetic\_gene\_cluster | 27 | 38.9623601221 | 133 | 1.1e-30 |
| AAM54090.1 | halogenase | BGC0000020 | Ansamitocin\_biosynthetic\_gene\_cluster | 26 | 38.4537131231 | 133 | 1.4e-30 |
| AAM70353.1 | CalO3 | BGC0000033 | Calicheamicin\_biosynthetic\_gene\_cluster | 29 | 37.7416073245 | 132 | 3.2e-30 |
| ACE80255.1 | HrmQ | BGC0000374 | Hormaomycin\_biosynthetic\_gene\_cluster | 28 | 37.0295015259 | 132 | 3.2e-30 |
| AAQ90172.1 | putative\_halogenase | BGC0000128 | Pyoluteorin\_biosynthetic\_gene\_cluster | 26 | 37.4364191251 | 109 | 1.7e-23 |
| AAD24884.1 | putative\_halogenase | BGC0000127 | Pyoluteorin\_biosynthetic\_gene\_cluster | 26 | 38.2502543235 | 109 | 2.2e-23 |
| ESP90845.1 | Dehydrogenases\_(flavoprotein) | BGC0000891 | Bromophenols\_/\_bromopyrroles\_biosynthetic\_gene\_cluster | 25 | 36.2156663276 | 104 | 9.2e-22 |
| AHA34035.1 | Bmp2 | BGC0000890 | Bromophenols\_/\_bromopyrroles\_biosynthetic\_gene\_cluster | 25 | 36.2156663276 | 101 | 4.6e-21 |
| AGO86665.1 | equisetin\_N-methyltransferase | BGC0001255 | Equisetin\_biosynthetic\_gene\_cluster | 25 | 36.012207528 | 88 | 5.2e-17 |
| CCH26291.1 | putative\_O-methyltransferase | BGC0001158\_c2 | Bikaverin\_biosynthetic\_gene\_cluster | 22 | 44.1505595117 | 88 | 5.2e-17 |
| ABQ04143.1 | tryptophan\_halogenase | BGC0000838 | Flexirubin\_biosynthetic\_gene\_cluster | 24 | 35.3001017294 | 87 | 1.2e-16 |
